# COLONOSCOPY: RANDOMIZED COMPARATIVE STUDY OF INSUFFLATION WITH CARBON DIOXIDE VERSUS AIR

**DOI:** 10.1590/0102-6720201700030004

**Published:** 2017

**Authors:** Luiz Gustavo DE-QUADROS, Roberto Luiz KAISER-JÚNIOR, Valter Nilton FELIX, Lucio VILLAR, Josemberg Marins CAMPOS, Vinicius Quintiliano Moutinho NOGUEIRA, André TEIXEIRA, Idiberto José ZOTARELLI-FILHO

**Affiliations:** 1 Kaiser Clinic and Hospital, Colonoscopy and Endoscopy Service, São José do Rio Preto, SP, Brazil; 2 Federal University of Pernambuco, Department of Bariatric Surgery, Recife, PE, Brazil; 3 Faculty of Medicine of the ABC, Department of Endoscopy, Santo André, SP, Brazil; 4 Faculty of Medicine, University of São Paulo, Department of Endoscopy and Bariatric Surgery, São Paulo, SP, Brazil; 5 Clinical Health, Endoscopy and Bariatric Surgery Service, Orlando, Florida, USA

**Keywords:** Colonoscopy, Insufflation, Patient satisfaction, Clinical protocols

## Abstract

**Background:**

In Brazil, an increasing number of people are submitted to colonoscopy, either for screening or for therapeutic purposes.

**Aim:**

To evaluate whether there are advantages of using carbon dioxide (CO_2_) over air for insufflation.

**Methods:**

Two hundred and ten of 219 patients were considered eligible for this study and were randomized into two groups according to the gas insufflation used: Air Group (n=104) and CO_2_ Group (n=97). The study employed a double-blind design.

**Results:**

The Air and CO_2_ Groups were similar in respect to bowel preparation evaluated using the Boston scale, age, gender, previous surgery, maneuvers necessary for the advancement of the device, and presence of polyps, tumors or signs of diverticulitis. However, “waking up with pain” and “pain at discharge” were more prevalent in the Air Group, albeit not statistically significant, with post-exam bloating seen only in the Air Group. The responses to a questionnaire, applied to analyze the late post-exam period, showed more comfort with the use of CO_2_.

**Conclusions:**

The use of CO_2_ is better than air as it avoids post-examination bloating, thereby providing greater comfort to patients.

## RESUMO

**Racional:**

No Brasil, estima-se crescente aumento da população submetida à colonoscopia, apesar do desconforto do exame, decorrente sobretudo da insuflação colônica.

**Objetivo:**

Verificar se há vantagens do uso de CO_2_ sobre o ar como elemento de insuflação.

**Métodos:**

Um total de 219 participantes foram submetidos à análise de elegibilidade e dele extraíram-se 210 eleitos, que foram randomizados em dois grupos, de acordo com o elemento utilizado: ar, n=104 e CO_2,_ n=97. O ensaio seguiu o modelo duplo-cego.

**Resultados:**

Os grupos demonstraram-se similares quando cotejados preparo intestinal avaliado pela Escala de Boston, idade, gênero, operação prévia, manobras necessárias para progressão do aparelho, presença de pólipo, tumor ou sinais de diverticulite, valorizando a comparação entre eles quanto ao elemento de insuflação. Então, observou-se que “acordar com dor” e a presença de dor na ocasião da alta foram bem mais prevalentes no “Grupo Ar”, embora sem diferença estatisticamente significante, sendo a distensão pós-exame observada apenas no “Grupo Ar”. De acordo com o questionário clínico aplicado para análise do período tardio pós-exame, as respostas apontaram muito mais conforto com o uso do CO_2_. Os elementos de insuflação não pareceram modificar substancialmente os aspectos técnicos do exame nem provocar índices expressivos de enantema da mucosa.

**Conclusão:**

O uso do dióxido de carbono é superior ao ar, pois evita a distensão abdominal pós-exame conferindo maior conforto aos pacientes no período pós-exame.

## INTRODUCTION

According to the World Health Organization, about 20 million colonoscopies are performed annually in the United States, primarily for colorectal cancer (WHO, 2016). In Brazil, an increase in the population submitted to colonoscopy is estimated, although inadequate preparation is still a major problem, leading to a repetition of the procedure (IBGE-Brazil, 2016). Due to this growing demand, it is imperative initially to carry out adequate preparation, making it possible to identify more accurately and precociously colorectal cancer, one of the main causes of death in the world, with an incidence of 900,000 cases per year^20^. On the other hand, for the patient, it is also relevant to perform a procedure with little discomfort, adequate sedation and analgesia, and to have a post-exam without pain or distension^22^. New techniques have been applied in order to reduce the discomfort during and after the examination^22^. This resulted in the use of carbon dioxide (CO_2_) for insufflation. It diffuses 160 times faster than air, allowing it to be more rapidly absorbed and excreted through respiration, decreasing the chance of spasm and pain^8^ ^,^ ^22^. However, air insufflation has remained the most widely used technique for distension of colonic lumen since the advent of colonoscopy in the 1960s, although it is always considered one of the causes of discomfort during and after it^12^ ^,^ ^19^. In this sense, the use of carbon dioxide insufflation could be beneficial to the patient, even though no perforations have been observed with its use, despite some reports of the complication in computerized colonographies^18^ ^,^ ^26^. Moreover, studies in which it was used, and in which arterial blood gas analysis was performed before and after colonoscopy, registered insignificant increases in pCO_2_, without altering the pH^3^ ^,^ ^21^. There are still no reports of adverse events even in patients with chronic obstructive pulmonary disease^8^.

It is worth mentioning three clinical trials comparing CO_2_ and air insufflation.

The first distributed 247 patients randomly into two groups: 124 for air and 123 for carbon dioxide. As a result, there was significantly less abdominal pain 10 min after colonoscopy in the group in which carbon dioxide was used^8^

The second study compared CO_2_ and air as an insufflation agent in 120 patients, 66 for CO_2_ and 54 for air submitted to colonoscopy without sedation. The CO_2_ volume used during the examination (14 l) was much higher than the previously reported mean of 8.3 l, but nevertheless, there was less pain and less time of examination in relation to the group in which it air was used^26^.

The third, based on PubMed, compared all randomized clinical trials between 1952 and 2008, encompassing 813 patients. As a result, abdominal pain (p<0.05), pain duration (p<0.05), and post-examination abdominal distension (p<0.05) were significantly reduced with the use of CO_2_ instead of air^1^.

To date, the use of CO_2_ as an insufflation element in the colonoscopy is not universalized, which opens space for this research, whose objective was to verify if it actually has advantages over air use, in the light of the analysis of technical variables related to the examination and clinical questionnaire to assess patient comfort during and up to 24 h post-procedure.

## METHODS

Participants were submitted to the eligibility analysis, followed by the CONSORT (Randomized controlled trials) rules. The trial followed the double-blind model, with all exams performed by a single colonoscopist. The study was evaluated and approved by the Research Ethics Committee of Beneficência Portuguesa Hospital under number 655.036 on May 19, 2014, in São José do Rio Preto, SP, Brazil.

### Participants

Eligibility was defined as individuals aged 14-90 years, with more than three bowel movements per week on previous days, excluding those with pregnancy, acute abdomen, hemorrhoids or recent endoscopic procedures, history of cancer, toxic megacolon, toxic colitis, idiopathic pseudo-obstruction, outlet obstruction, severe fecal retention, peptic ulcer, gastroparesis, ileus, previous operation of the upper gastrointestinal tract. Other factors of exclusion were angina and/or myocardial infarction in the last three months, congestive heart failure and uncontrolled arterial hypertension, renal insufficiency or known hypersensitivity to elements to be used in the preparation or sedation.

To verify similarity between the groups, age, gender, quality of bowel preparation, using the Boston Scale^6^, previous operation of the lower gastrointestinal tract, need for postural and compressive maneuvers to progression of the device (hereinafter referred to as “maneuver”), presence of polyp, tumor, and signs of diverticulitis. For the comparison between use of CO_2_ or air in the insufflation, the patients were observed clinically after the examination, valuing the complaints of pain as soon as they awoke and of pain and sensation of abdominal distension at discharge. They were asked to respond to a questionnaire, through the Internet, to report pain intensity, the need to use medication or the assistance of the team during a 24 h period after discharge, and to assess their degree of comfort and satisfaction with the examination . Assigning values ​​to each response, according to Table 1, it was possible to analyze each response by software created in the SurveyMonkey® platform.

**TABLE 1:** Verification of similarity between groups - gender, age, quality of bowel preparation (Boston scale)

	Age(years)	Gender	Boston Scale
CO_2_	Average: 48 (±15) Mín: 16 Max: 83	Men: 30 Women: 67	6.75
p	0.09	0.14	0.73
AIR	Average: 44 (±15) Mín: 14 Max: 80	Men: 46 Women: 58	7.50

Patients were clinically observed from the time they woke up until discharge, and the complaint of pain was valued, as well as the sensation of abdominal distension. They were asked to respond to a questionnaire, to report pain intensity in the post-procedure, in the clinic and at home, need to resort to the assistance of the team during pain or discomfort, and to assess their quality of life. 

In addition to the presence or absence of mucosal enanthema in the progressive removal of the device, technical aspects of the examination also counted in the comparison between the groups: the time of arrival to the ileum and the time of withdrawal of the colonoscope.

### Interventions

The exams were indicated either for screening or for diagnostic clarification. Before the procedure, the patient ingested four Dulcolax^®^ tablets, with tea or water in the morning, liquid diet (broth, juice, tea or water) at lunch, two 25 mg Dramin^®^ capsules in the afternoon, sodium picosulfate, a 12 g sachet dissolved in 150 ml of cold water, followed by five 250 ml cups of water and other liquids until midnight, whereupon absolute fasting was instituted until colonoscopy was performed in the morning. Sedation was made with propofol, 40 mg/dose until reaching the appropriate level.

### Outcomes

The first focus was on the efficacy of the CO_2_insufflation technique in relation to air insufflation for a subsequent colonoscopic procedure.

The second, analyzed the patients through an internet questionnaire, asking the intensity of pain, the need to use medication or the assistance during a period of 24 h after discharge, and to evaluate their degree of comfort and satisfaction with the exam.

### Randomization and double-blind assay

After patient´s enrollment, they were numbered and randomized. Each number was randomly assigned to a group until the end of the number of patients. It was conducted by one professional not participating in the study. 

### Sample size and recruitment

A total of 219 participants were selected, and 210 were randomized into two groups after signature of the Consent Term, according to the colonoscopy insufflation element: air (n=104) and CO_2_ (n=97).

In the follow-up phase, only 62 patients in the CO_2_ group completed the questionnaire and 44 in the air.

After randomization, the participants were recruited in each group to perform the procedures. These were double-blind, that is, neither the principal investigator nor the patient knew the type of examination applied.

The recruitment period began after the signature of the Term of Consent by all the participants that were included in the present study. The follow-up of each patient started during the procedure itself, once the comfort or pain was analyzed during each examination. In addition, follow-up continued at the end of the procedure, in the immediate periods - at home and after 24 h. Patients who actually participated in the follow-up were asked to complete a questionnaire.

### Statistical analysis

Kolmogorov-Smirnov normality test was performed for continuous and categorical data, and Kruskal-Wallis variance analysis for non-parametric and Spearman variables for parametric variables. Logistic regression was used for categorical variables and linear regression for continuous variables. For all the tests was adopted alpha level of 0.05. Linear regression was also tested by the presence of continuous predictors and response predictors, as well as Durbin-Watson residue analysis, with p<0.0025, adopting as an acceptable range of independence 1.69 <dw <2.31. In addition to the attention for the presence or absence of mucosal enanthema on the progressive removal of the device, technical aspects of the examination also were used in the comparison between the groups: the time of arrival to the ileum and the time of withdrawal of the colonoscope.

## RESULTS

### Primary results

As shown in Tables 1 and 2, there was no statistically significant difference between the groups, when compared to age, gender, intestinal preparation (evaluated by the Boston Scale), previous operation, maneuver, presence of polyp, tumor or signs of diverticulitis. Regarding the technical aspects of the examination (Table 3), the time of arrival to the ileum was about 3 min in both, while the colonoscope withdrawal was higher in the CO_2_ group, with p<0.05.

**TABLE 2:** Similarity check between groups: previous operation, maneuver, polyp, tumor and signs of diverticulitis

	Previous operation	Maneuver	Polyp	Tumor	Diverticulitis
CO_2_	Yes=68 (70) No=29 (30)	Yes=7 (7) No=90 (93)	Yes=34 (35) No=63 (65)	Yes=1 (1) No=96 (99)	Yes=1 (1) No=96 (99)
p	0.23	0.33	0.05	0.05	0.23
Air	Yes=54 (52) No=50 (48)	Yes=13 (13) No=88 (91)	Yes=34 (33) No=70 (67)	Yes=5 (5) No=99 (95)	Yes=9(9) No=95(91)

**TABLE 3:** Comparison between use of CO_2_ or air in the insufflation: presence of mucosal enanthema and technical aspects of the examination

Group	Enanthema	Arrival time (min)	Withdrawn time(min)
CO_2_	Yes=3 (3%) No=94 (97%)	Mean: 3.1 (±2) Min: 1 Max: 13	Mean: 8.3 (±5.5) Min: 3 Max: 43
P	0.330	0.137	0.038*
Air	Yes=7 (7%) No=97 (93%)	Mean: 2.6 (±1.4) Min: 1 Max: 8	Mean 7.9 (±3.6) Min: 2 Max: 25

### Secondary results

Table 4 shows that “waking up with pain” is much more prevalent in the Air group, as well as the presence of pain at discharge, although a statistically significant difference was not achieved. On the other hand, the sensation of abdominal distension was not noticed in the CO_2_ Group and present in 16% of the cases in the Air group. There were 62 completed questionnaires in the CO_2_ group and 44 in the Air and the answers, compiled in Table 5, point much more comfort with the use of CO_2_, although without statistical expression. Few patients were symptomatically treated, and less than 5% of each group used the team’s services. “Very good” comfort degree was achieved in about 63% of CO_2_ group, while no more than 35% in the Air group. There was no difference in relation to the presence of mucosal enanthema in both groups.

**TABLE 4:** Comparison between use of CO2 or air in the insufflation

	Wake up with pain	Pain at discharge	Distention
CO_2_	Yes=1 (1%) No=99 (99%)	Yes=2 (2%) No=96 (98%)	No=97 (100%)
p	0.693	0.560	0.05
Air	Yes=17 (16%) No=87 (84%)	Yes=18 (17%) No=86 (83%)	Yes=17 (16%) No=87 (84%)

**TABLE 5:** Comparison between use of CO_2_ or air in the insufflation: answers to the clinical questionnaire

Group	Intensity of pain in the clinic	Pain at home (24 h post-procedure)	Use of medication at home	Needed medical care	Degree of comfort after the exam
CO_2_	Painless=86.44 %	Painless=70.97%	Painless=88.71%	No=95.16%	Very good=62.90%
	Little=10.17%	Little=19.35%	Little=3.23%	Yes=4.84%	Good=25.81%
	Moderate=3.39%	Moderate=3.23%	Moderate=4.84%		Medium=4.84%
	Strong=0.00%	Strong=6.45%	Strong=3.23%		Bad=4.84%
	Intense =0.00%	Intense =0.00%	Intense =0.00%		Very bad = 1.61%
p	0.46 0.46 0.25 0.05 0.05	0.12 0.25 0.25 0.05 0.05	0.678 0.555 0.05 0.55 0.99	0.96 0.96	0.37 0.37 0.59 0.05 0.69
Air	1)Painless=75.00%	Painless=45.45 % Little=36.36% Moderate=15.91% Strong=0.00% Intense=2.27%	Painless=90.91 % Little=6.82 % Moderate=0% Strong=2.27% Intense =0%	No=95.45% Yes=4.55%	Very Good=34.09% Good=56.82% Medium=6.82% Bad= 0% Very bad=2.27%
	2)Little=4.55 %				
	3)Moderate=13.64%				
	4) Strong=4.55 %				
	5)Intense=2.27 %				

### Auxiliary analyses

**FIGURE 1:**
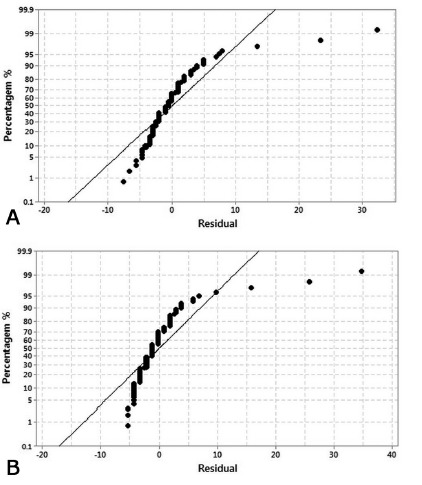
A) Linear regression of CO2 results; B) linear regression of air resuls

In order to deepen the exploratory results, after the linear regression test between the predictors response and continuous predictors for both CO_2_ and air, it was observed that for some cases the results were significant, that is, they presented interdependence, with p<0.05 (With “p=0.05”) between “Ileum vs. Maneuver Time”, “Ileum vs. Polyp Time”, “Ileum vs. Gender Time”, “Withdrawal Time vs. Polyp”, “Withdrawal Time vs. Age” and “Withdrawal Time vs. Gender”. For Air, the results were significant for “Woke up with Pain vs. Tumor”, “Ileum vs. Preoperative Time”, “Ileum vs. Maneuver Time”, “Ileum vs. Age”, “Ileum Time Vs. Gender”,” Withdrawal Time vs. Polyp” and “Withdrawal Time vs. Tumor “(Figure 1).

In addition, more sensitive statistical treatment was performed by the dependence relationship analysis (autocorrelation) of Durbin-Watson Statistic residues. Therefore, in cases where the null hypothesis (H0) has not been rejected, there was no autocorrelation, that is, the residuals were independent with a 95% confidence level. In this sense, all cases in the CO_2_ group were independent, that is, there was no significant relation between the residues. In the Air group, there was a dependence relationship between the predictors “Pain at discharge vs. Comorbidities”, “Pain at discharge vs. Maneuver”, “Pain at discharge vs. Polyp”, “Withdrawal Time vs. Tumor” and “Withdrawal Time vs. DII”; thus, in these cases, there was a significant relationship between the residues, rejecting the null hypothesis (H0).

### Unwanted results

For the CO_2_ and Air groups, significant damages or unwanted effects were “waking pain”, “pain on discharge”, “enanthema” and “distension”, but these undesirable effects were more evident in the Air group.

## DISCUSSION

Rigid eligibility criteria facilitated the homogenization of the groups, which were similar, regarding age, gender, previous operation, maneuvers necessary for the progression of the device, presence of polyp, tumor or signs of diverticulitis, as shown statistically, therefore adequate for CO_2_ or air insufflation comparison in colonoscopy, with the least possible undesirable interference. It was even taken care that the sedation did not provide analgesia, hence the choice of propofol as an anesthetic agent.

The results presented in Tables 3 and 4 support the greater tendency of the literature to affirm the advantage of CO_2_ as an element of colonic insufflation for colonoscopy in relation to air. The use of CO_2_ was initially proposed by Becker in 1953, but it was not until the 1980s that endoscopists began to consider CO_2_ insufflation as a potential method to reduce pain after colonoscopy^11^.

Similar studies were done^7,9,17^ until in a recent meta-analysis^22^ that showed lower prevalence of post-colonoscopy abdominal pain after use of CO_2_, in comparison with those who had air, regardless of having undergone the examination under moderate, deep sedation, or even without sedation. The same result was reported in a study that included 214 ileocolonoscopies, conducted under sedation with propofol^15^.

Another study model with abdominal radiographies being performed from 30 min to 6 h after colonoscopy, brought up the information that there is less intestinal gas in patients receiving CO_2_insufflation^11^ ^,^ ^23^.

It has been highlighted that abdominal distension is rare with the use of CO_2_ ^4^ ^,^ ^5^ ^,^ ^10^ ^,^ ^13^ ^,^ ^14^ ^,^ ^16^, which, in fact, was noticed in this series, in which there was no case of distension in the CO_2_ group, against 16% in air. Maeda et al. (2013) also found no difference between the CO_2_ and air, regarding cecal intubation rates, cecal intubation times or total time of examination. Previous studies have described a greater depth of intubation with the use of CO_2_ ^24^ ^,^ ^25^.

In this series, the time of arrival to the ileum was independent of the insufflation element, as well as the presence of enanthema of the mucosa. The longer withdrawal time in the CO_2_ group does not seem to have any real relation to the blowing element, at least apparently, but this must be related to the peculiarities of each examination.

The differences in the majority of the analyzes did not statistically substantiate the advantage of using CO_2_ as an inflation factor, except for post-examination distention; but, it is relevant that assessing pain at different moments and post-exam comfort always favored CO_2_, at the expense of air use.

As limitations of the present study, technical aspects of colonoscopy do not seem to be influenced by the insufflation element, but the results stimulate the continuation of the study with greater number, since perhaps this can lead to statistical significance of the differences. Research on the cost-effectiveness and safety profile of CO_2_ insufflation can be done in patients with chronic obstructive pulmonary disease.

## CONCLUSION

The use of carbon dioxide as an insufflation element in the colonoscopy avoids post-examination abdominal distension, which seems to be superior to the use of air also for pain upon waking of the examination and after discharge, conferring a greater degree of comfort to the patients submitted to the procedure, since from the immediate post-exam time to 24 h after the procedure.
